# Application of data mining techniques and logistic regression to model drug use transition to injection: a case study in drug use treatment centers in Kermanshah Province, Iran

**DOI:** 10.1186/s13011-019-0242-1

**Published:** 2019-12-12

**Authors:** Somayeh Najafi-Ghobadi, Khadijeh Najafi-Ghobadi, Lily Tapak, Abbas Aghaei

**Affiliations:** 1 Department of Industrial Engineering, Factually of Engineering, Kermanshah Branch, Islamic Azad University, Kermanshah, Iran; 2 Department of Biostatistics, School of Public Health, Hamadan University of Medical Sciences, Hamadan, Iran; 3 Department of Biostatistics, School of Public Health, Modeling of Noncommunicable Diseases Research Center, Hamadan University of Medical Sciences, Hamadan, 65175-4171 Iran; 4 PhD in Epidemiology, Social Determinants of Health Research Center, Research Institute for Health Development, Kurdistan University of Medical Sciences, Sanandaj, Iran

**Keywords:** Drug injection, Neural network, Decision tree, Support vector machine, Logistic regression

## Abstract

**Background:**

Drug injection has been increasing over the past decades all over the world. Hepatitis B and C viruses (HBV and HCV) are two common infections among people who inject drugs (PWID) and more than 60% of new human immunodeficiency virus (HIV) cases are PWID. Thus, investigating risk factors associated with drug use transition to injection is essential and was the aim of this research.

**Methods:**

We used a database from drug use treatment centers in Kermanshah Province (Iran) in 2013 that included 2098 records of people who use drugs (PWUD). The information of 29 potential risk factors that are commonly used in the literature on drug use was selected. We employed four classification methods (decision tree, neural network, support vector machine, and logistic regression) to determine factors affecting the decision of PWUD to transition to injection.

**Results:**

The average specificity of all models was over 84%. Support vector machine produced the highest specificity (0.9). Also, this model showed the highest total accuracy (0.91), sensitivity (0.94), positive likelihood ratio [1] and Kappa (0.94) and the smallest negative likelihood ratio (0). Therefore, important factors according to the support vector machine model were used for further interpretation.

**Conclusions:**

Based on the support vector machine model, the use of heroin, cocaine, and hallucinogens were identified as the three most important factors associated with drug use transition injection. The results further indicated that PWUD with the history of prison or using drug due to curiosity and unemployment are at higher risks. Unemployment and unreliable sources of income were other suggested factors of transition in this research.

## Background

Drug injection has been increasing over the past decades all over the world [2]. Compared to smoking, inhaling, snorting and swallowing, injecting of drugs for various reasons, like non-compliance with health tips, increases the chance of health consequences such as viral infections. Using shared needles and syringes spread infectious diseases among people who inject drugs (PWID). High prevalence rates of HBV and HCV among PWID represent the vulnerability of this population [3]; the chance of HCV infection is 53 times higher among PWID compared with general population [4]. According to the results of a meta-analysis related to the incidence time of HCV infection (considering from the onset of injection), the one-year cumulative incidence of drug injection was 28% (with 95% CI: 17–42%) [5].

Recently, a systematic review of HIV among people who use drugs (PWUD) showed that the prevalence of HIV among PWID is 4.4 times more than others [6]. A third of all HIV cases outside of sub-Saharan Africa are PWID [7]. Also, this infection can spread to other groups of society via sexual relationships with PWID. In seven out of ten areas under the coverage of the joint United Nations’ program on HIV and AIDS (UNAIDS), drug injection was identified as the first (or second) cause of HIV transmission [8, 9].

It is estimated that there are approximately 260,000 PWID in Iran [10], and more than 60% of new HIV cases are PWID.

Iran has adopted large-scale harm reduction policies such as provision of methadone maintenance treatment (MMT) and needle and syringe programs targeting PWID since 2002. Although these policies are the most important preventive measure against drug injection and risks experienced by PWID [11], it is believed that preventing injection initiation takes precedence over reducing a range of risks that these individuals encounter with after starting the use of drug injection [12–14].. Experiences in Amsterdam, Netherlands, and New York, USA, [15, 16] showed that preventing the transition to drug injection is quite feasible. However, little attention has been paid to the prevention of PWUD to transition from other routes of drug administration (smoking, inhaling, snorting and swallowing) to injection in Iran. A better understating of risk factors associated with drug use transition to injection in Iran can help authorities make more effective preventive strategies and identify PWUD at risk of transition. This research aimed to determine these factors, using classification models.

It should be noted that the performance of different classification models may vary over different datasets. No model works very well in all situations. Therefore, we employed the most widely used classifiers (neural network and support vector machine, decision tree and logistic regression) whose prediction accuracy has been confirmed by several studies [17–19]. At final, the model with the best performance was used to interpret the findings.

## Methods

### Dataset

**Fig. 1:**
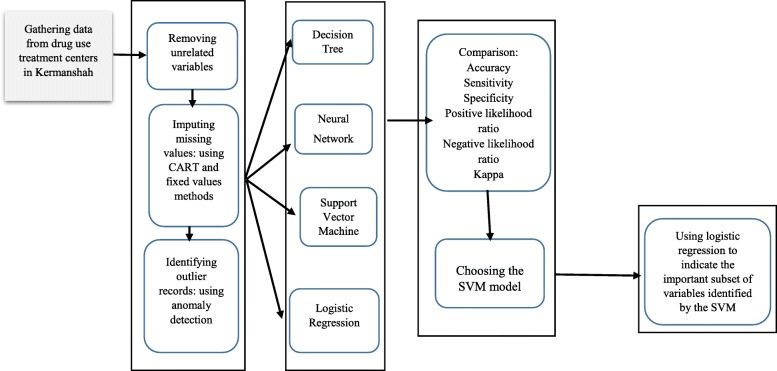
Classification model building process.

This research used a dataset that included 2098 records. The data were collected based on a researcher-made checklist of information about people who were referred to drug use treatment centers. The checklist was completed by the PWUD, therapist or experts and consultant of treatment centers. Based on agreement with the treatment centers, checklists were collected based on specific codes for each individual and personal information (such as name, family and national code) was not included in the checklists. Informed consent was obtained from the PWUD to permission of using the data and permission to do this research had been registered with the Ethics Committee of Kermanshah University of Medical Sciences under code KUMS.RES.1394.480. Our methodology for modeling process are shown in Fig. 1.

We used the information of 29 risk factors that are believed associate to transition PWUD to injection. These risk factors included, age, gender, marital status, housing status, education, occupational status, age at the first drug use experience, the first used drug, number of years of drug use, family history of drug use, history of suicide, history of overdose, history of mental disorder of the individual and the family, history of taking opium, hallucinogens, hashish, heroin, sap (the milky latex sap of opium), crystal, cocaine, amphetamine, sedative, methadone, cigarette and alcohol, history of prison, number of referrals to drug use treatment centers, motivation for starting drug use. History of drug injection was considered as dependent variable with two subsets: people who inject drugs (PWID) and people who do not inject drugs (people who smoky, inhale, snort or swallow drugs) (PWNID). For cases with a history of injection, only those were enrolled that injection was the latest type of drug using.

### Data pre-processing and dealing with missing values

Before model application, the missing data and outliers were checked consistently. The missing data across all variables for the dataset ranged from 0 to 11.83%. The highest missing data were history of suicide (11.83%) and history of overdose (1.24%). The data for these variables were imputed by using CART regression trees. CART is one of the popular methods for imputing missing data. It was proposed by Breiman et al. in 1984 [20]. The other missing data with missing values lower than 0.057% (history of mental disorder of the individual and the family, history of prison, marital status, housing status, history of drug injection, number of referrals to drug treatment centers, and motivation for starting drug use) were imputed by their mode. Anomaly detection was used for finding the outlier records. Anomaly detection provides very significant and critical information for outlier detection in various applications [21]. Fifteen records with anomaly index greater than 2 [22] were eliminated from further investigations. The eliminated records were belonged to PWNID that was the majority class. So, deleting these records because they were outliers did not affect the results.

The variable of housing status encompassed four subsets of home ownership, rentals, homelessness, and others. Furthermore, the homelessness and others were merged as one group. Marital status was defined as married, divorced or widow (widower) and single. Since in more than 80% of cases, the first used drug was the family of opium (opium and sap), then the first used drug variable was divided into the opioids and other drugs. In order to facilitate the interpretation of the results, university degrees of associate, bachelor, and master were combined to one single group of “College education” to analyze the variable of education (with no record in the PhD group). Occupational status was reduced into four groups of unemployed, self-employed, employed and housewife. The motivation variable for first drug use including factors such as sex enhancement, drugs available and others were merged into one single group. The demographic and summary statistics of variables included in the analysis for the full dataset were shown in Tables 1 and 2. For cases with a history of injection, only those enrolled that injection was the latest type of drug using.

**Table 1:** Summary of discrete variables

Variables	PWNID		PWID	
	N	Percentage	N	Percentage
Gender				
Women	59	0.03	3	0.01
Men	1765	0.97	256	0.99
Marital status				
Married	1252	0.69	108	0.42
Widow	126	0.07	45	0.17
Single	446	0.24	106	0.41
Occupational status				
Unemployed	238	0.13	88	0.34
Self-employed	1241	0.68	159	0.61
Employed	297	0.15	9	0.03
Housewife	48	0.03	3	0.01
Housing status				
Home ownership	1326	0.73	196	0.76
Rentals	480	0.26	57	0.22
Homeless	18	0.01	6	0.02
Education				
Illiterate	98	0.05	3	0.01
Elementary	259	0.14	28	0.11
Leadership	543	0.3	108	0.42
High School	685	0.38	102	0.39
College education	239	0.13	18	0.07
The first used drug				
Opioids	1614	0.88	172	0.66
Others	210	0.12	87	0.34
Motivation for starting the drug use				
Pleasure	823	0.45	78	0.30
Drug use of friends	441	0.24	83	0.32
Curiosity	132	0.07	32	0.12
Use as a pain reliever	92	0.05	21	0.08
Emotional distress and mental	141	0.08	9	0.04
Others	96	0.05	12	0.05
Unemployment	99	0.05	24	0.09
Family history of drug use				
No	1194	0.65	154	0.60
Yes	630	0.35	105	0.40
History of taking opium				
No	260	0.14	50	0.19
Yes	1564	0.86	209	0.81
History of taking hallucinogens				
No	1809	0.99	235	0.91
Yes	15	0.01	24	0.09
History of taking crystal				
No	1608	0.88	148	0.57
Yes	216	0.12	111	0.43
History of taking heroin				
No	1547	0.85	78	0.30
Yes	277	0.15	181	0.70
History of taking hashish				
No	1621	0.89	142	0.55
Yes	203	0.11	117	0.45
History of taking sap^*^				
No	981	0.54	138	0.53
Yes	843	0.46	121	0.47
History of taking cocaine				
No	1716	0.94	192	0.74
Yes	108	0.06	67	0.26
History of taking sedative				
No	1695	0.93	205	0.79
Yes	129	0.07	54	0.21
History of taking amphetamine				
No	1812	0.99	259	1
Yes	12	0.01	0	0
History of taking methadone				
No	1704	0.93	226	0.87
Yes	120	0.07	33	0.13
History of taking cigarette				
No	350	0.19	25	0.1
Yes	1474	0.81	234	0.9
History of taking alcohol				
No	1606	0.88	169	0.65
Yes	218	0.12	90	0.35
History of overdose**				
No	1632	0.89	225	0.87
Yes	192	0.11	34	0.13
History of suicide				
No	1724	0.94	190	0.73
Yes	100	0.06	69	0.27
History of mental disorder				
No	1587	0.87	205	0.79
Yes	237	0.13	54	0.21
History of mental disorder in family				
No	1749	0.96	232	0.90
Yes	75	0.04	27	0.1
History of prisons				
No	1473	0.81	84	0.32
Yes	351	0.19	175	0.68
Number of referrals to drug treatment centers				
1	167	0.09	7	0.03
2	493	0.27	42	0.16
3	376	0.21	30	0.12
4	470	0.26	69	0.27
5	318	0.17	111	0.43

*The milky latex sap of opium

** He/ she experienced overdose

**Table 2:** Summary of continues variables

Variables	PWNID					PWID				
	N	Min	Max	Mean	Std. Dev	N	Min	Max	Mean	Std. Dev
Age	1824	17	90	38.77	11	259	21	61	34.34	8.11
Age at the first drug use experience	1824	17	62	23.64	7.05	259	8	42	19.40	5.23
Number of years of drug use	1824	0.5	50	12.23	9.09	259	2	38	12.84	7.77

### Classification models

Decision tree, neural network, support vector machine and logistic regression were employed to identify factors affecting PWUD‘s decisions to shift to injection among the people who were referred to the treatment centers for drug use in Kermanshah in 2013.

Decision trees (DTs) fit piecewise constant models by recursively partitioning the predictor spaces [23]. They are helpful in identifying individuals with or without history of injection through easily interpreted grouping rules. A rule is induced by a binary split on covariates with questions such as “Has the history of taking heroin” or “Is the subject male or female?” According to some criteria, the algorithm searches for the best split among all possible splits and the data are partitioned accordingly. The procedure is repeated till the data set is split into a number of mutually exclusive groups. Decision tree is simple to understand and interpret even with hard data. Although it is unstable and with a small changing in data, the optimal decision tree change very large.

The field of neural networks (NNs) was originally kindled by psychologists and neurobiologists who sought to develop and test computational analogues of neurons [24]. Roughly speaking, an NN is a set of connected input/output units in which each connection has a weight associated with it. During the learning phase, the network learns by adjusting the weights so as to be able to predict the correct class label of the input tuples. NNs involve long training times, and are, therefore, more suitable for applications where long training time is feasible. It requires a number of parameters that are typically best determined empirically, such as the network topology or “structure”. Several topologies of NNs can be used in binary classification problems. Two of the most commonly used NNs are the Multilayer Perceptron (MLP) and the Radial Basis Function (RBF). The main differences between these two NNs reside in the activation functions of the hidden layers. NN has the ability to model a dataset with a large number of input variables and highly complex nonlinear relationships. The disadvantage of NN is that this is a “black box” and output cannot be explicitly interpreted [25–27].

Support vector machine (SVM) is based on the fact that with an appropriate function to a sufficiently high dimension, data from two categories can always be separated by a hyperplane [28]. SVM separates a given set of binary labeled training data with a hyperplane that is maximally distant from them (known as the maximal margin hyper-plane). Data are then classified according to which side of the hyperplane they lie on. SVM model provides efficient solutions to classification problems without considering any assumption about the distribution of data and models nonlinearity of the variables based on minimization of structural risk [18]. The main disadvantage of the SVM is that there are several key parameters such as Kernel function that should be set correctly to attain the best results for any particular problem.

Logistic regression (LR) is a standard statistical Generalized Linear Model (GLM) approach for modeling binary outcomes [29]. In this approach, the logit of the conditional probability of dependent variable (history of drug injection) being formulated as a linear function of independent variables. The slope parameters in a logistic model can be interpreted as a log of odds ratios. Simple linear structure, widely available fitting software and some flexibility to deal with categorical variables are the main advantages of LR. However, the LR method is sensitive to dependent variables and the researcher must choose them correctly before using it.

All the models were fitted with the variables introduced in Tables 1 and 2. 70% of the data was used as training data and 30% as testing data. The IBM SPSS modeler 14.2 was applied for data analysis.

### Imbalanced dataset

Our dataset was imbalanced because the data for PWNID and PWID were 1824 and 259, respectively. Imbalanced data set creates a new challenging problem for data mining models, because standard classification algorithms usually consider a balanced training set and this makes a bias towards the majority class. So, a number of solutions to the class-imbalance problem were previously proposed both at the sampling and algorithmic levels [30]. At the sampling level, these solutions include many different forms of re-sampling such as random oversampling, random under-sampling, and combination of them. Random under-sampling seeks to create balance between the two classes by reducing the size of the majority class. This is accomplished by randomly removing instances from the majority class until the desired class ratio has been achieved. Alternatively, random oversampling seeks to improve the class balance by increasing the size of the minority class. The increase is performed through randomly duplicating instances from the minority class until the desired class ratio has been achieved [31]. At the algorithmic level, solutions include adjusting the costs of the various classes so as to counter the class imbalance, and adjusting the probabilistic estimate at the tree leaf (when working with decision trees). In this research, a combination of oversampling and under-sampling methods were used for NN and LR. For DT method, combination of oversampling and under-sampling methods and cost method were used. Since the result for the SVM without considering the class-imbalance problem was acceptable, therefore, we did not consider the imbalanced problem for the SVM model.

### Implementation and performance criteria

For comparing the models, we used 10-fold cross-validation: one with 90% subjects for training and the other with 10% subjects for validation. This process repeated 10 times. Then, Sensitivity, specificity, total accuracy, positive likelihood ratio, negative likelihood ratio and Kappa were used to compare the models and calculated based on the following formulas:

$$ Sensitivity=\frac{TP}{TP+ FN}, Specificity=\frac{TN}{TN+ FP}, Total\kern0.17em Acuraccy=\frac{TP+ TN}{TP+ FP+ TN+ FN} $$

$$ Positive\ likelihood\ ratio=\frac{Sensitivity}{1- Specificity} $$

$$ Negetive\ likelihood\ ratio=\frac{1- Sensitivity}{Specificity} $$

$$ Kappa=\frac{P_o-{P}_e}{1-{P}_e}\kern3.12em {P}_o=\frac{TP+ TN}{TP+ FP+ TN+ FN}\kern3em {P}_e=\frac{\left( TP+ TN\right)\left( TP+ FN\right)+\left( FN+ TN\right)\left( FP+ TN\right)}{{\left( TP+ FN+ TN+ FP\right)}^2} $$

Where TP, FP, TN, and FN represent the number of true positives, false positives, true negatives, and false negatives, respectively. Classification models indicate the importance of a variable based on the percentage increase in the prediction error. A variable is selected as the most important if it creates the most error when it is removed. After scoring the importance of variables, they are ranked based on their importance.

## Results

### Data mining models

#### Decision tree

**Fig. 2:**
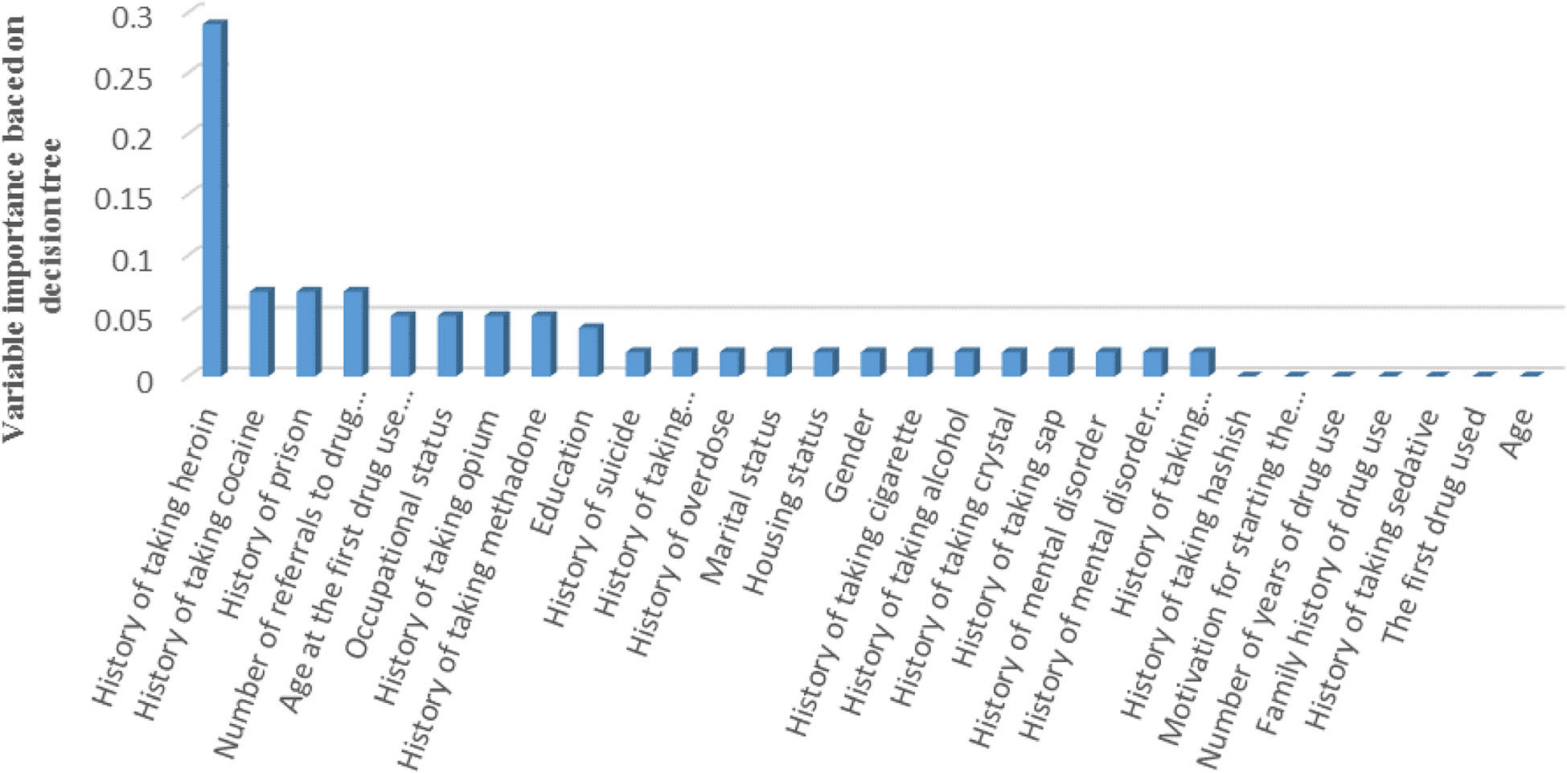
Importance of variables estimated by the decision tree

The number of variables in this research was large. Therefore, we used C5.0 decision tree that can automatically winnow the variables before a classifier is constructed, discarding those that appear to be only marginally relevant. This algorithm generates smaller classifiers with higher predictive accuracy, and can often reduce the time required to generate rule sets. The decision tree (DT) was created with three different methods: a) combination of oversampling and undersampling methods, b) cost method, and c) combination of the first and second methods. Different settings of the parameters were tested, and the best result was obtained by the first method. The samples of PWNID and PWID were multiplied by 0.6 and 4 for the training samples, respectively. Expected noise was set zero. Also, simple and accuracy were used for mode and favor in the software, respectively. The most informative variables, according to the values of variable importance, estimated by the DT model were shown in Fig. 2.

#### Neural network

**Fig. 3:**
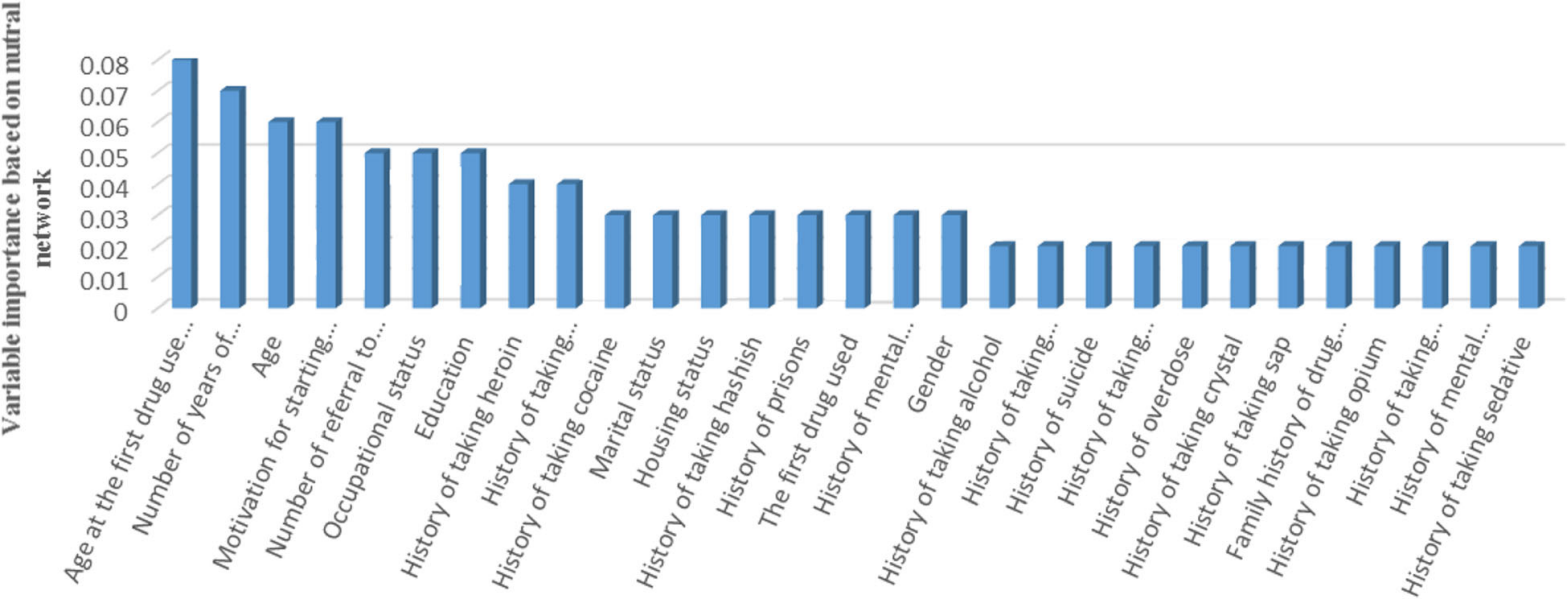
Importance of variables estimated by the neural network

In this research, the multilayer perceptron was trained with 30 inputs (one for each predictor) in the input layer and two hidden layers with 30 and 18 neurons. The number of neurons in the hidden layer was iteratively adjusted by the software to minimize classification errors in the training dataset. Maximum training time and overfit prevention were set 15 min and 30%, respectively. Figure 3 showed the importance of variables associated with drug injection by the NN model.

#### Support vector machine

**Fig. 4:**
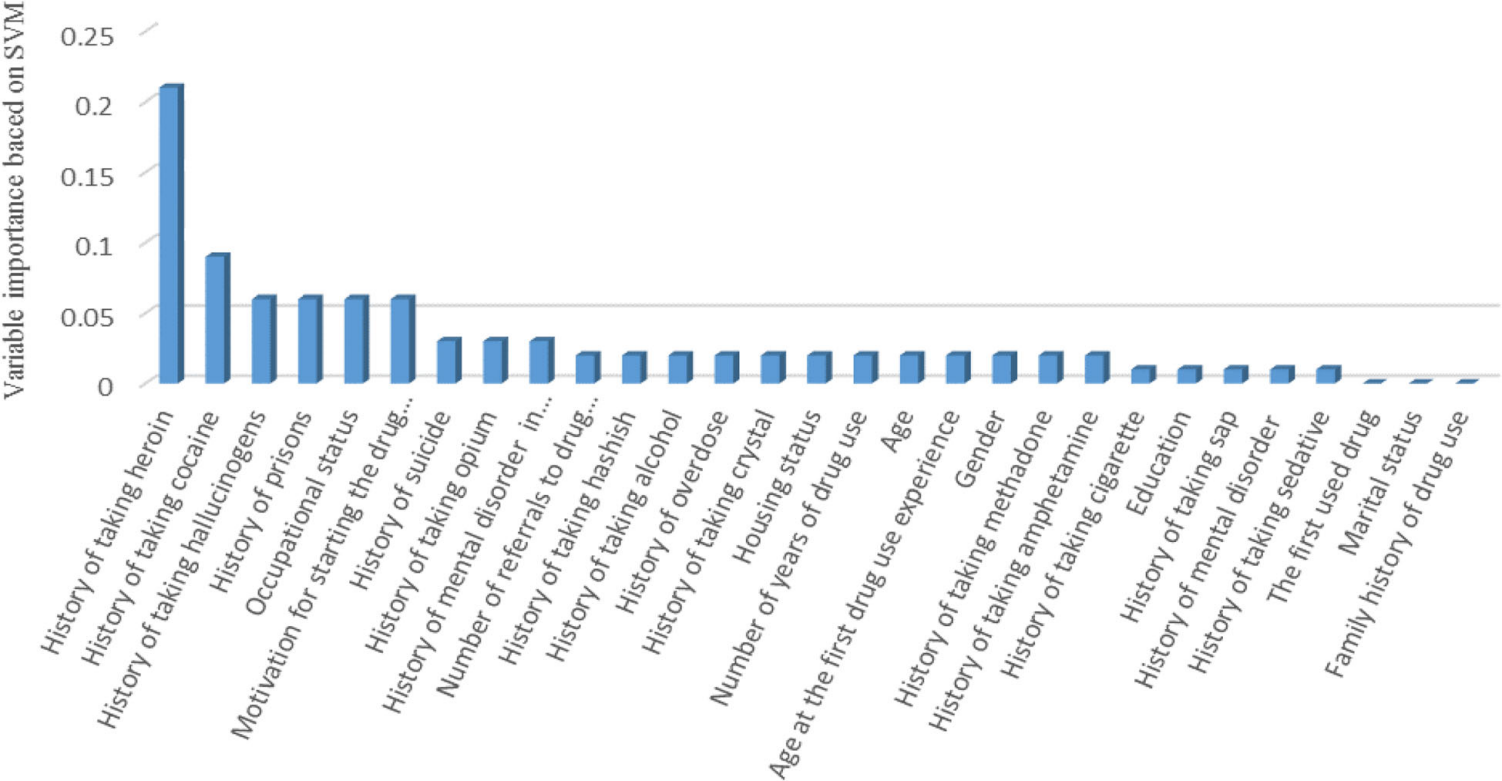
Importance of variables estimated by the support vector machine

The polynomial function was used as kernel for the SVM model because it had better results than other kernel functions in our dataset. Regularization (C) and degree parameters were optimized by trying different values, and the best-obtained values were 15 and 3, respectively. We used expert mode and stopping criteria was set 0.001. The SVM model ranked all of the variables associated with drug injection, and the final results were shown in Fig. 4.

#### Logistic regression

Based on *p* <  0.05, the backwards stepwise logistic regression (LR) model indicated occupational status, education, the first used drug, number of years of drug use, motivation for starting drug use, number of referrals to drug treatment centers, family history of drug use, history of taking heroin, history of taking hashish, history of taking cocaine, history of taking hallucinogens, history of taking crystal, history of taking methadone, history of suicide, history of prison, as statistically significant factors associated with drug injection (Table 3). Reference subset was “*having no history of injection*”.

**Table 3:** Logistic regression model

Variables	Odds ratio (OR)	95% confidence interval for OR		Wald	*P*-value
Occupational status					
Unemployed	–	–	–	–	–
Self-employed	0.548	0.134	2.232	0.705	0.401
Employed	0.303	0.075	1.218	2.831	0.092
Housewife	0.090	0.017	0.477	8.006	0.005
Education					
Illiterate	–	–	–	–	–
Elementary	0.202	0.044	0.926	4.239	0.040
Leadership	0.438	0.183	1.050	3.421	0.064
High School	0.919	0.459	1.838	0.057	0.811
College education	0.905	0.467	1.754	0.087	0.767
The first used drug					
Opioids	–	–	–	–	–
Others	0.417	0.275	0.633	16.909	< 0.001
Number of years of drug use	1.047	1.021	1.075	12.390	< 0.001
Motivation for starting the drug use					
Pleasure	–	–	–	–	–
Drug use of friends	0.592	0.271	1.293	1.729	.0.188
Curiosity	1.592	0.725	3.498	1.341	0.247
Use as a pain reliever	2.207	0.929	5.244	3.215	0.073
Emotional distress and mental	0.000	0.00		0.000	0.996
Others	0.760	0.287	2.014	0.304	0.581
Unemployment	0.492	0.167	1.454	1.647	0.199
Family history of drug use					
yes	1. 403	0.938	2.099	2.712	0.100
History of taking hashish					
yes	0.446	0.299	0.665	15.694	< 0.001
History of taking heroin					
yes	0.107	0.070	0.164	106.375	< 0.001
History of taking cocaine					
yes	0.165	0.101	0.270	51.905	< 0.001
History of taking hallucinogens					
yes	0.059	0.024	0.146	37.579	< 0.001
History of taking crystal					
yes	0.674	0.451	1.008	3.684	0.055
History of taking methadone					
yes	0.487	0.252	0.941	4.591	0.032
History of suicide					
yes	0.353	0.220	0.566	18.650	< 0.001
History of prison					
yes	0.594	0.394	0.897	6.143	0.013
Number of referrals to drug treatment centers					
1	–	–	–	–	–
2	0.291	0.101	0.835	5.271	0.022 0.032 0.001
3	0.556	0.326	0.950	4.608	
4	0.381	0.217	0.670	11.235	
> 5	0.604	0.384	0.949	4.780	0.029

### Model comparison

Table 4 showed the total accuracy, sensitivity, specificity, positive likelihood ratio, negative likelihood ratio (Mean and standard deviation) and Kappa estimated by the cross-validation of the testing set for each models. The results indicated that the reliability indices of SVM model were higher than the other three models.

**Table 4:** Mean and standard deviation of total accuracy, sensitivity, specificity, positive likelihood ratio, negative likelihood ratio and Kappa statistic for DT, NN, SVM and LR

Models	Total accuracy		Sensitivity		Specificity		Positive likelihood ratio		Negative likelihood ratio		Kappa
	Mean	Std. dev	Mean	Std. dev	Mean	Std. dev	Mean	Std. dev	Mean	Std. dev	
Decision tree	0.82	0.043	0.76	0.027	0.86	0.025	25.27	12	0	0	0.87
Neural network	0.83	0.02	0.87	0.031	0.84	0.021	17	7.67	0.027	0.03	0.79
Support vector machine	0.91	0.01	0.94	0.017	0.90	0.022	35	15	0	0	0.94
Logistic regression	0.65	0.025	0.74	0.095	0.85	0.026	5.78	1.18	0.19	0.09	0.48

### Applying logistic regression to important variables of the SVM model

The SVM model delineates the important variables but does not show which subset of these variables are significant. For this reason, we modeled a logistic regression based on six major variables as independent variables that had importance greater than 0.05 (including history of taking heroin, history of taking cocaine and history of taking hallucinogens, history of prison, motivation for starting drug use, and occupational status) and history of drug injection as dependent variable. Reference subset was “*having no history of injection*”. The obtained results were shown in Table 5.

**Table 5:** Logistic regression model based on the six important variables of the SVM model

Variables	Odds ratio (OR)	95% confidence interval for OR		*P*-value
Occupational status (reference = Housewife)				
Unemployed	1.495	0.40	5.54	0.547
Self-employed	0.782	0.22	2.83	0.708
Employee	0.362	0.84	1.57	0.174
Motivation for starting the drug use (reference = unemployment)				
Pleasure	0.483	0.25	0.94	0.032
Drug use of friends	0.932	0.48	1.81	0.836
Curiosity	1.478	0.70	3.13	0.307
Use as a pain reliever	0.122	0.09	0.21	< 0.001
Emotional distress and mental	0.634	0.27	1.49	0.297
Others	0.324	0.12	0.89	0.029
History of taking heroin				
Yes	12.23	8.33	17.81	< 0.001
History of taking cocaine				
Yes	5.92	3.71	9.48	< 0.001
History of taking hallucinogens				
Yes	19.01	43.38	8.24	< 0.001
History of prison				
Yes	2.43	1.70	4.35	< 0.001

Table 5 showed that the odds ratio of being unemployment to housewife was 1.495 more in transition to drug injection. Also, the odds ratio of being self-employed and employed to housewife were 0.782 and 0.362 lower in transition to drug injection, respectively. Results revealed that having the history of prison and history of taking heroin, hallucinogens, and cocaine are another important factors. Our findings indicated that the odds ratio of people who start to use drugs because of curiosity to unemployment was 1.478 more in transition to injection. The odds ratio of people who start to use drugs because of pleasure, drug use of friends, curiosity, emotional distress and mental, use as a pain reliever and others to unemployment were lower than 1.

## Discussion

This research aimed at determining risk factors associated with transition to injection among the PWUD referred to drug use treatment centers in Kermanshah Province in 2013, using logistic regression, decision tree, natural network and support vector machine. Based on the reliability indices, the SVM model outperformed other models. Therefore, this model was used for further interpretation.

Our finding indicated unemployment as a risk factor associated with drug use transition to injection. This result is consistent with the findings of Abelson et al. 2006 [32]. They expressed that unreliable source of income was a determining factor in transition to injection. Results of the SVM further showed that the history of taking heroin, hallucinogens, and cocaine are another important factors. It is noticeable that the decision tree model also predicted histories of taking heroin and cocaine as the most important variables. Harocopos et al. (2009) and Neaigus et al. (2006) reported that many PWNID used heroin and cocaine before injection [16, 33]. Rahimi et al. (2012) believed that heroin and opium were the predominant patterns of drug use before the first injection [34]. Also, Cheng et al. (2006) stated that the rate of transition to injection use in Iran and other countries in the Middle and South Asia, with the higher rates of heroin use among PWNID, was higher than in the areas with higher use of stimulants [35].

Hallucinogens are new addiction substances that like heroin and cocaine provide different sense in PWUD in comparison to traditional substances (opium and sap). The hallucinogenic substance was not identified in previous researches; therefore, it was added to our research.

In the present research, having the history of prison was another factor identified as effective in transition to injection. Since injection is smokeless and odorless, imprisoned PWUD prefer it in prison. Low availability, poor quality, and high cost of drugs are the main factors that facilitate the transition to injection in prison [1]. This finding is in line with the results from studies conducted in other developing countries [1, 35–37]. Carles (2005) found that imprisonment increased the probability of transition to injection [37]. Between 6 and 48% of prisoners injected drugs throughout their lives [38].

The variable of motivation for starting drug use has not been considered in previous researches; therefore, it was added to our research. Our results showed that people who start to use drugs because of curiosity are at higher risk in transition to injection.

### Limitations

There were some limitations in this research. First, this study was a cross-sectional study and therefore the temporality relationship between case and outcome cannot be properly approved, but as cases with a history of injection, only those enrolled in study that injection were the latest type of drug using, that can be said that these findings can greatly right. Second, in this research, we selected potential risk factors associated with drug use transition to injection from the literature of drug use. There may be other factors not mentioned in the literature that we could identify by interviewing experts.

## Conclusion

The aim of this research was to identify risk factors associated with drug use transition to injection, employing four classification methods (decision tree, neural network, support vector machine, and logistic regression).

According to the findings, it was concluded that the heroin, cocaine and hallucinogenic substances can play an effective role in transition of PWUD to injection. Efforts to reduce the use of these substances in society should be more increased. Also, those who use them should be more supported and monitored as being more susceptible to transition to injection. PWUD with a history of imprisonment are another group at risk. The entrance and exit channels of prison should be further scrutinized to prevent the entry of drugs into prison. Also, in prisons, policymakers provide treatment services for PWUD.

With respect to drug using, since unemployment and unreliable sources of income are important factors, creating jobs for PWUD is essential.

## Data Availability

The data is available to authors.

## Abbreviations

CART: Classification and Regression Trees
DT: Decision tree
HBV: Hepatitis B virus
HCV: Hepatitis C virus
HIV: Human immunodeficiency virus
LR: Logistic regression
MMT: Methadone maintenance treatment
NN: Neural network
PWID: People who inject drugs
PWNID: People who do not inject drugs (people who smoky, inhale, snort or swallow drugs)
PWUD: People who use drugs
SVM: Support vector machine
